# The Book World of Medicine and Science

**Published:** 1902-07-12

**Authors:** 


### The Book World of Medicine and Science.

Poison Romance and Poison Mysteries. By C. J. S. Thompson, F.R.Hist.S. New and revised Edition. (London: The Scientific Press.)

Those interested in toxicology will welcome this new and extended edition of Mr. Thompson's book on poison mysteries and romance. The poisonings of the past are more attractive as stories than are those of to-day. Many people who derive their information from fiction take an exaggerated view of the cunning and chemical knowledge of the poisoners of the Middle Ages. Their success, however, lay more in the igrorance of others than in any special knowledge of their own. As regards the poison romances of the dark ages, many of which are so lucidly set forth in the volume now before us, the agents employed were poisons which would now be almost inevitably detected by a chemist's assistant. Of the science of toxicology little or nothing was then known, and the subjects of disease and medicine were so hedged round by ignorance and superstition, that even ordinary maladies were not infrequently attributed to the malevolent practices of sorcery. Small wonder, then, that in such an age the crime of poisoning should so easily have escaped detection. During the fifteenth and sixteenth centuries poisoning seems to have played a prominent part in European politics. In Italy of that period it was a commonplace of party politics, and became so universal as to be a constant source of dread and danger to those in authority. Alluding to this, Mr. Thompson tells how, in 1543, John of Raguba, a Franciscan brother, exhibited to the Venetian Council of Ten a selection of choice poisons, declaring himself ready to remove by such means any person held politically obnoxious by them. His terms were concise and business-like. A pension of 1,500 ducats per annum on the death of the first victim, and the sum to be increased upon the successful execution of fresh orders. The matter was solemnly discussed, and the offer finally accepted by the council, who further decided that the Emperor Maxmillian would be the most suitable object of John's first operations.

The most common forms of poison at that time were belladonna, nux vomica, aconite, hellebore, arsenic, and opium, these being frequently introduced into the winecup. The modern poisoner would hold such death-dealing as little less clumsy than mere bludgeoning, so accurately has the presence of these poisons come to be diagnosed from the symptoms of the victim, or by means of chemical analysis before or after death. Arsenic, from quite early times until recent years, has ever been a favourite weapon in the hands of the female poisoner. Among the more modern poisoning cases narrated by Mr. Thompson are included the celebrated mystery of Madeleine Smith, who, in 1857 was accused of poisoning her lover by means of arsenic, and the more recent case of Mrs. Maybrick, charged with poisoning her husband.

A Text-Book of Insanity. By Charles Mercier, M.B., M.R.C.P., F.R.C.S. (London: Swan, Sonnenschein and Co. 1902. Price 6s. net.)

For the student who wishes, in small compass and without entering deeply into psychological problems, to obtain a useful working knowledge of those varieties of insanity which he is likely to meet with in practice, this little book by Dr. Mercier is admirably suited. The key to Dr. Mercier's method of teaching is to be found in his insistance on the fact that it is by conduct that a man must be judged. As he says, "what goes on in the minds of other people we can never know unless and until it is revealed by conduct," but when conduct is disordered we may safely infer the existence of insanity without stopping to investigate the condition of his mind. This point he aptly illustrates by saying that "if a general officer goes on parade in flannels and practising the banjo; if a parson goes into the pulpit and plays cup and ball before the congregation; if a hostess comes down to a dinner party in her nightdress and curl-papers . . . . we need not sit down and investigate the state of their minds before we pronounce them insane; the state of the mind is left on one side, and does not enter into our consideration." On the other hand there are cases in which an investigation of the state of the mind is most important, but these are cases in which a portion, it may be a very large portion, of the conduct exhibits no disorder. The state of the mind has then to be investigated in order that one may discover whether conduct is likely to exhibit disorder. But if we discover a disorder of mind that has no influence upon conduct, we cannot regard it as an indication of insanity. After a discussion of what he terms the Institutes of Insanty, Dr. Mercier proceeds to a description of the forms and varieties of insanity. It is here that the originality and utility of his classification adopted show themselves to the greatest advantage. Casting aside the older divisions of the subject he describes the various forms of disordered conduct which may arise, and then goes on to describe the several varieties of insanity which are met with. Thus we get rid of the old division of insanity into mania, melancholia, etc., which really represent only temporary phases in the history of very different maladies, and instead find typical groupings of symptoms and successions of symptoms which may be taken as describing so many different "diseases" of the mind, if we like to use such a term. By a "form" of insanity Dr. Mercier means a certain aggregate of symptoms that a case of insanity presents at one time; while by a "variety," he means a specific course that a case may run from beginning to end, usually combined with an assignable cause. Thus, the same variety may at different times run through several forms—may begin with excitement, after which it may take on the form of systematised delusions, and later again may take on the form of simple weakmindedness. On the other hand, the same form of insanity may be met with in several distinct varieties. For example, depression appears in acute insanity, in general paralysis, in circular insanity, in climacteric insanity, and in other varieties. This is a most practical way of dealing with the subject. The following are the varieties of insanity which are described:—idiocy and imbecility, dementia, stupor, acute delirious mania, acute insanity, fixed delusion, paranoia, folie circulaire, insanity of reproduction, insanity of times of life, insanity of alcohol, general paralysis, insanity of epilepsy, and insanity of bodily disease. The symptoms and the course of these several types of insanity are set forth in a very lucid manner.

